# Long-term gamma-aminobutyric acid (GABA) treatment fails to regain beta-cell function in longstanding type 1 diabetes in a randomized trial

**DOI:** 10.1038/s41598-025-95751-y

**Published:** 2025-04-04

**Authors:** Henrik Hill, Per Lundkvist, Georgios Tsatsaris, Bryndis Birnir, Daniel Espes, Per-Ola Carlsson

**Affiliations:** 1 Department of Women’s and Children’s Health, Uppsala University, Uppsala, Sweden; 2 Department of Medical Sciences, Uppsala University, Uppsala, Sweden; 3 Department of Medical Cell Biology, Uppsala University, Uppsala, Sweden; 4 Science for Life Laboratory, Uppsala University, Uppsala, Sweden

**Keywords:** Type 1 diabetes, GABA, Beta-Cell, Oral therapy, Regenerative therapy, Type 1 diabetes, Phase II trials

## Abstract

Gamma-amino butyric acid (GABA) has in experimental studies been found to promote beta-cell proliferation, enhance insulin secretion and reduce inflammation, positioning it as a candidate drug for type 1 diabetes (T1D) therapy. This phase I/II randomized controlled trial assessed the safety and efficacy of long-term treatment with Remygen^®^ (Diamyd Medical), a controlled-release oral GABA formulation, as a potential beta-cell regenerative therapy in adults with long-standing T1D. Thirty-five male subjects with T1D (≥ 5 years) were randomized into three arms receiving the study drug(s) once daily for 6 months: GABA 200 mg (Arm 1), GABA 600 mg (Arm 2) and GABA 600 mg + alprazolam 0.5 mg for 3 months followed by GABA 600 mg alone for 3 months (Arm 3). Safety measures, hormonal counter-regulation during hypoglycemic clamps, fasting- and stimulated C-peptide levels, were assessed at multiple timepoints. Safety concerns included elevated aspartate aminotransferase (AST) in nine subjects, leading to the withdrawal of two subjects. Most elevations were, however, transient with no dose-differences. No effects were observed on fasting- or stimulated C-peptide levels, CGM metrics or HbA1c. Hypoglycemic hormonal counter-regulation was unaltered. To conclude, we found no clinical evidence of a beta-cell regenerative effect of GABA, but side effects were commonly observed.

**Supplementary Information:**

The online version contains supplementary material available at 10.1038/s41598-025-95751-y.

## Introduction

Type 1 diabetes (T1D) is characterized by an immune-mediated destruction of the beta-cells within the islets of Langerhans in the pancreas^1^. Already upon diagnosis, T1D patients have typically lost a majority of their beta-cell mass and function, which then further decline over time^2,3^. However, even decades after debut, some endogenous beta-cell function commonly remains^4–6^. These observations open for the possibility to target these cells that have been spared by the immune system with regenerative drugs aiming to induce beta-cell proliferation. Even a partial regain of beta-cell function would be of utmost clinical value, since residual insulin production has been found to reduce both the acute and long-term complications associated with T1D^7,8^.

Gamma-aminobutyric acid (GABA) is a well-known signaling mediator within the central nervous system (CNS), exerting its effect through ionotropic GABA-A and metabotropic GABA-B receptors. Outside of the CNS, GABA is found in the highest concentrations within immune cells and pancreatic beta-cells^9,10^. Since GABA is synthesized from glutamate through the enzymatic action of glutamic acid decarboxylase (GAD), an enzyme to which auto-antibodies commonly form during the development of T1D, the physiological role of GABA in the immune system and pancreatic islets has gained interest. Immunologically, GABA seems to exert immunosuppressive effects by decreasing the production of pro-inflammatory cytokines and increasing regulatory T-cells (Treg)^11,12^. Furthermore, GABA has been found to dampen both the inflammatory process and beta-cell death in murine models of T1D and in human islets^13–15^. Endocrinologically, GABA has been identified to promote insulin secretion and stimulate beta-cell proliferation in both mouse and human islets^14,16,17^. Furthermore, GABA has also been attributed to induce transdifferentiation of pancreatic ductal cells to insulin-producing beta-cells via glucagon-producing alpha-cells^18,19^. In experimental studies, these beta-cell proliferative effects of GABA have been synergistically increased by coadministration of alprazolam, acting as a positive allosteric modulator (PAM) of the GABA-A receptor^20,21^. Beyond beta-cell proliferation, GABA has also been reported to stimulate beta-cell neogenesis, even in the absence of remaining beta-cells, through the transdifferentiation of other cell types^19^. However, it is important to note that this concept has been challenged, as attempts to replicate these findings in experimental studies have proven difficult^22,23^. Despite variable outcomes in experimental studies, the clinical evaluation of GABA in T1D remains essential.

We recently proved in a phase I clinical trial that the drug candidate Remygen^®^, an orally administered controlled-release formulation of GABA, is well tolerated as a short-term therapy and that it can establish a counter-regulatory response to hypoglycemia in patients with long-standing T1D^24^.

In the present clinical phase I/II trial, we have assessed the safety and efficacy of Remygen^®^ during an extended time period (6 months) as a potential beta-cell regenerative drug for the treatment of long-standing T1D.

## Research design and methods

### Ethical statement

The study was a phase I/II, 3-arm, open label, single center study investigating the safety and effect of oral GABA therapy on beta-cell regeneration in long-standing T1D. GABA was manufactured as Remygen^®^, since GABA is classified as a pharmaceutical drug in the European Union. Participants were provided oral and written study information and signed a written consent prior to inclusion. The study was conducted in accordance with the Declaration of Helsinki of 1964 and its subsequent amendments and approved by both the Swedish Ethical Review Authority (Dnr 2018/200) and the Swedish Medical Products Agency (EudraCT No. 2018-001115-73). Complete information of study design is presented at ClinicalTrials.gov (NCT03635437, registration date 17/08/2018).

### Study participants and study design

The study visits were conducted at Uppsala University Hospital, Uppsala, Sweden. Study participants were recruited through advertising. T1D patients aged ≥ 18 and ≤ 50, with T1D onset before 25 years of age and a disease duration of ≥ 5 years with C-peptide levels in the range from non-detectable up to < 0.12 nmol/L were qualified for screening. Females of child-bearing potential and males without adequate contraception were excluded from the study, since there are no studies of the teratogenic potential of GABA. For full inclusion- and exclusion criteria, see ClinicalTrials.gov (NCT03635437).

**Fig. 1:**
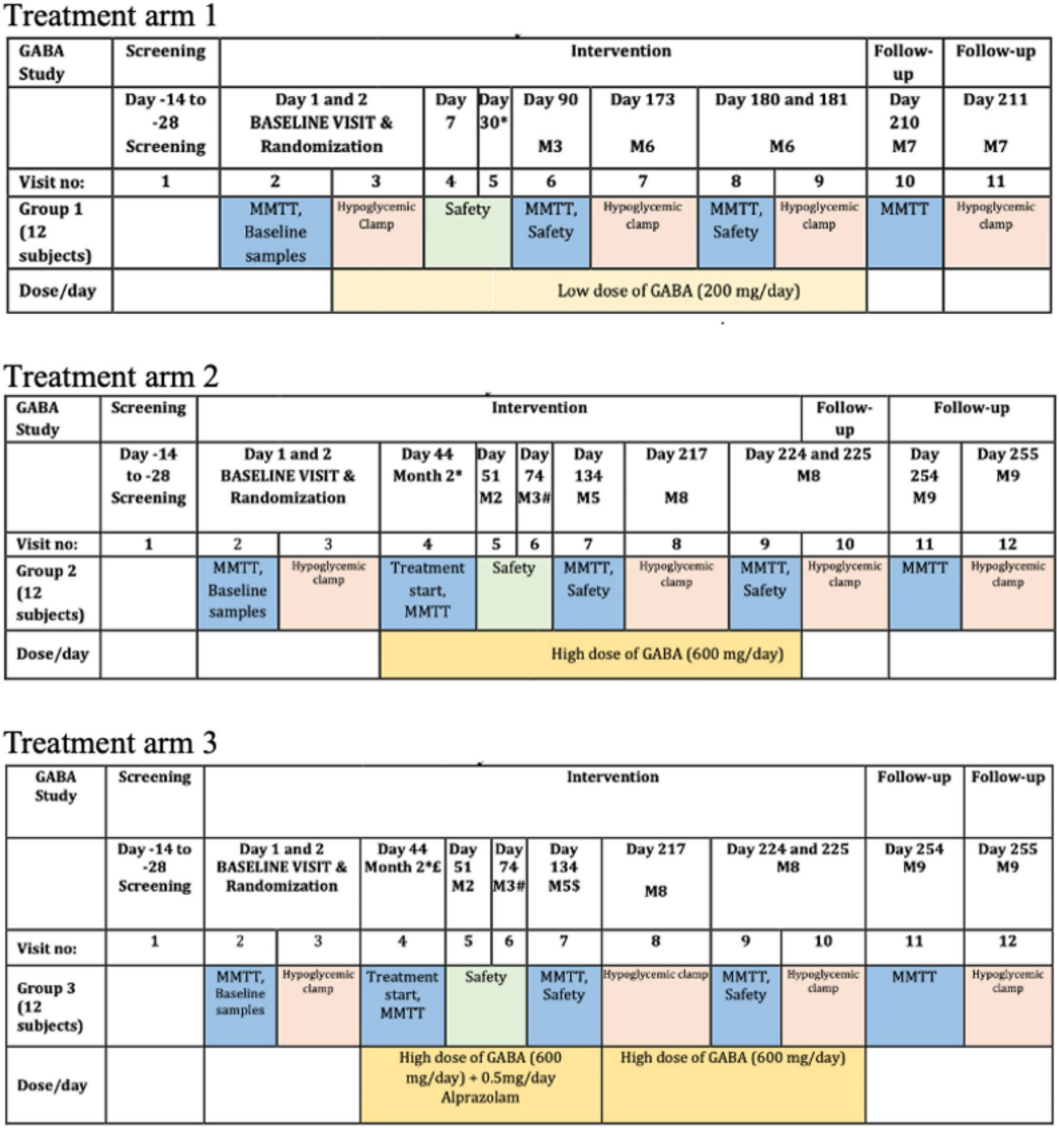
Study design and scheduled clinical visits in the three treatment groups. *DSMB evaluated the safety data of the first 4 subjects included in treatment arm 1, whereafter arms 2 and 3 were allowed to start. ^£^Treatment was initiated on day 44 with 0.5 mg of alprazolam. It was ramped up on day 45 with low-dose GABA (200 mg) and 0.5 mg of alprazolam, and further on day 46 with high-dose GABA (600 mg) and 0.5 mg of alprazolam. As an extra precaution, subjects remained under observation at the hospital for 3 h after tablet intake on days 44–46. ^#^DSMB evaluated the safety of the first 4 subjects in arms 2 and 3 to ensure no safety concerns had arisen upon treatment with the higher dose of GABA (and alprazolam in arm 3). ^$^Treatment with alprazolam stopped after 3 months, whereafter subjects continued with GABA alone for the remaining 3 months.

GABA was administered orally, once daily, in a controlled-release formulation, Remygen^®^ (for pharmacokinetics data, see Espes et al., 2021^24^), which has been developed by Diamyd Medical (Diamyd Medical, Stockholm, Sweden). Included study participants were randomized according to a randomization list produced by a statistician in a 1:1:1 ratio to receive 200 mg of GABA for 6 months (*Arm 1*), 600 mg of GABA for 6 months (*Arm 2*), or alprazolam 0.5 mg combined with 600 mg of GABA for 3 months followed by treatment with 600 mg of GABA alone for another 3 months (*Arm 3*). The initiation of high dose GABA treatment (i.e. *Arm 2* and *3*) was delayed until a Data Safety Monitoring Board (DSMB) had evaluated and approved the safety data from the first four included participants in *Arm 1*. Randomization was stratified by C-peptide levels to ensure that an equal number of subjects with detectable C-peptide were included in each arm. A visual summary of the study design and scheduled visits is presented in Fig. 1.

### Safety monitoring

All participants continued to receive their normal standard of care intensive insulin treatment from their personal physicians during the whole study period. In accordance with the study protocol, participants underwent safety assessments including occurrence of adverse events (AEs), general physical examinations, neurological examinations and laboratory safety assessments at several time points during the study (outlined in Fig. 1). Uppsala Clinical Research Center monitored the study, and data were reviewed by an external DSMB.

### Mixed meal tolerance test (MMTT)

Prior to the MMTT the participants were instructed to fast overnight (≥ 10 h). Participants on insulin therapy with multiple daily injections (MDI) were instructed to administer their long-acting insulin on the pre-ceding evening but not the same morning as the MMTT. Participants with continuous subcutaneous insulin infusion (CSII) continued with their basal rate throughout the test. In the case of CSII with automatic insulin delivery (AID) based on sensor glucose readings, the AID function was deactivated during the MMTT. No bolus dose of fast acting insulin was to be administered within 6 h before the test. Plasma glucose levels were targeted in the range of 4–12 mmol/L in the morning of the test, if outside of this range the test was re-scheduled. Prior to the test a venous catheter was inserted, from which blood samples were collected at baseline (time 0) and at 15, 30, 60, 90 and 120 min after oral intake of Resource protein (Nestlé Health Science, Switzerland) which was consumed within ≤ 5 min. The intake amount was calculated based on body weight (6 ml/kg, with a maximum dose of 360 ml). An MMTT was conducted at four occasions in *Arm 1* and at five occasions in *Arm 2* and *3 (*Fig. 1*)*. The daily dose of the study drug(s) was given following finalization of the MMTT’s.

### Hypoglycemic clamp

After an overnight fast (≥ 10 h), an individualized insulin infusion dose based on body weight (2mIU x kg-1 x min-1) was administered. A separate glucose infusion of 100 mg/ml was administered at various infusion rates to achieve and maintain initial normoglycemia (5.5 mmol/L) for 30 min, followed by hypoglycemia (2.5 mmol/L) for 30 min. Blood samples for the measurement of counter regulatory hormones, as described below, were collected when a stabile blood glucose concentration had been established at both of these target levels. The insulin infusion was thereafter terminated and the study participant observed until normoglycemia was achieved. Hypoglycemic clamps were performed at four occasions. The daily dose of the study drug(s) was administered after completion of the hypoglycemic clamp, except for the hypoglycemic clamp at treatment day 173, when the study drug was administered 20 min prior to initiation of the hypoclamp.

### Laboratory measurements

Laboratory safety assessments included hematology (mean corpuscular hemoglobin, mean corpuscular volume, mean corpuscular hemoglobin concentration, hemoglobin, platelets, total leukocyte count and differential count), clinical chemistry and liver function tests (alanine aminotransferase, aspartate aminotransferase, alkaline phosphatase, bilirubin). Urine analysis was performed to investigate presence of microalbuminuria. Metabolic parameters, including HbA1c, glucose, C-peptide and blood lipids (total cholesterol, HDL cholesterol, LDL cholesterol and triglycerides), were assessed under fasting conditions. GAD and IA2 antibodies were measured utilizing the standard clinical assay. Counter-regulatory hormones (plasma levels of growth hormone, cortisol, glucagon, epinephrine and norepinephrine) were assessed during the hypoglycemic clamps. All above mentioned tests were analyzed at the clinical chemistry laboratory of Uppsala University Hospital, except for epinephrine and norepinephrine which were analyzed at laboratories in Karolinska University Hospital (Sweden) and Skåne University Hospital (Sweden). C-peptide concentrations were, in addition to the clinical assay, also analyzed with an ultrasensitive C-peptide ELISA with a lower detection limit of 1.17 pmol/L (Mercodia, Uppsala, Sweden)^4^.

### GABA measurements

At several time-points through the study, EDTA plasma was collected and stored at -70 °C for later analyses of GABA. The blood samples were taken as a trough level to measure the concentration of the drug right before the next dose. Analyses were performed by mass spectrometry at the Mass Spectrometry Based Metabolomics Facility at Uppsala University, with a validated protocol and method approved by the US Food and Drug Administration^25^.

### Assessment of quality of life

Quality of Life (QoL) was assessed with the RAND-36 questionnaire at baseline, at last day of study drug and at follow-up (for arms 2 and 3 an additional test was performed at treatment start).

### Continuous glucose monitoring

Glucose levels were monitored by continuous glucose monitoring (CGM) (FreeStyle Libre, Abbott Laboratories, Chicago, Illinois, USA) during a 14-day period before visits at baseline, 3 months of treatment, 6 months of treatment and at the follow-up visit. Standard CGM-metrics were reported; % Time in range (TIR; 3.9–10 mmol/L), % Time below range (TBR; <3.9 mmol/L) and % Time above range (TAR; >10 mmol/L).

### Statistical analyses

Statistical analyses were conducted by Uppsala Clinical Research Center using either parametric methods (ANOVA) or nonparametric methods (Wilcoxon). Categorical data (such as quality of life and safety outcomes) were compared using Fisher´s exact test or the exact Chi-Square. For isCGM-data, a nonparametric one-way ANOVA with Dunn´s multiple comparisons test was applied. For the AUC of ultrasensitive C-peptide values derived from the MMTT, delta values of counter regulatory hormones and GABA levels compared to the baseline visit, mixed-effect analyses with Dunnett´s multiple comparison test were applied (in order to handle missing values).

Results are presented as mean ± SEM, and p-values < 0.05 were considered statistically significant.

#### Estimation of sample size

The primary efficacy analysis assessed changes in C-peptide levels from a 2-hour MMTT. Each test provided six C-peptide measurements (0, 15, 30, 60, 90, and 120 min), categorized as (1) Below 0.05 nmol/l (undetectable), (2) between 0.05 and 0.2 nmol/l, and (3) above 0.2 nmol/l. Changes in these categories over time were summarized in a transition table. With six classifications per subject and 10 subjects per group, the analysis included 180 total observations. The probability of detecting treatment benefit was calculated based on binomial probabilities, assuming independent outcomes and identical success probabilities across observations. The study was designed to detect treatment benefits with at least 80% power when the true benefit rate was as low as 1.5%. Mixed generalized linear modeling accounted for individual variability, ensuring reliable estimates of therapeutic response.

This confirmed the study was sufficiently powered to detect treatment benefit even at low probabilities.

### Data sets

The Full Analysis Set (FAS) included all randomized subjects who received at least one dose of the study medication and had at least one post-baseline assessment along with a corresponding baseline measurement for any efficacy variable. The FAS was considered the primary analysis dataset and was used for all primary and secondary efficacy variables. The Completers Set consisted of all patients in the FAS who completed the corresponding study period and who had secondary efficacy data available.

The Safety set comprised all randomized subjects who received at least one dose of the study medication, with safety presentations based on this set.

## Results

### Subject characteristics

**Fig. 2:**
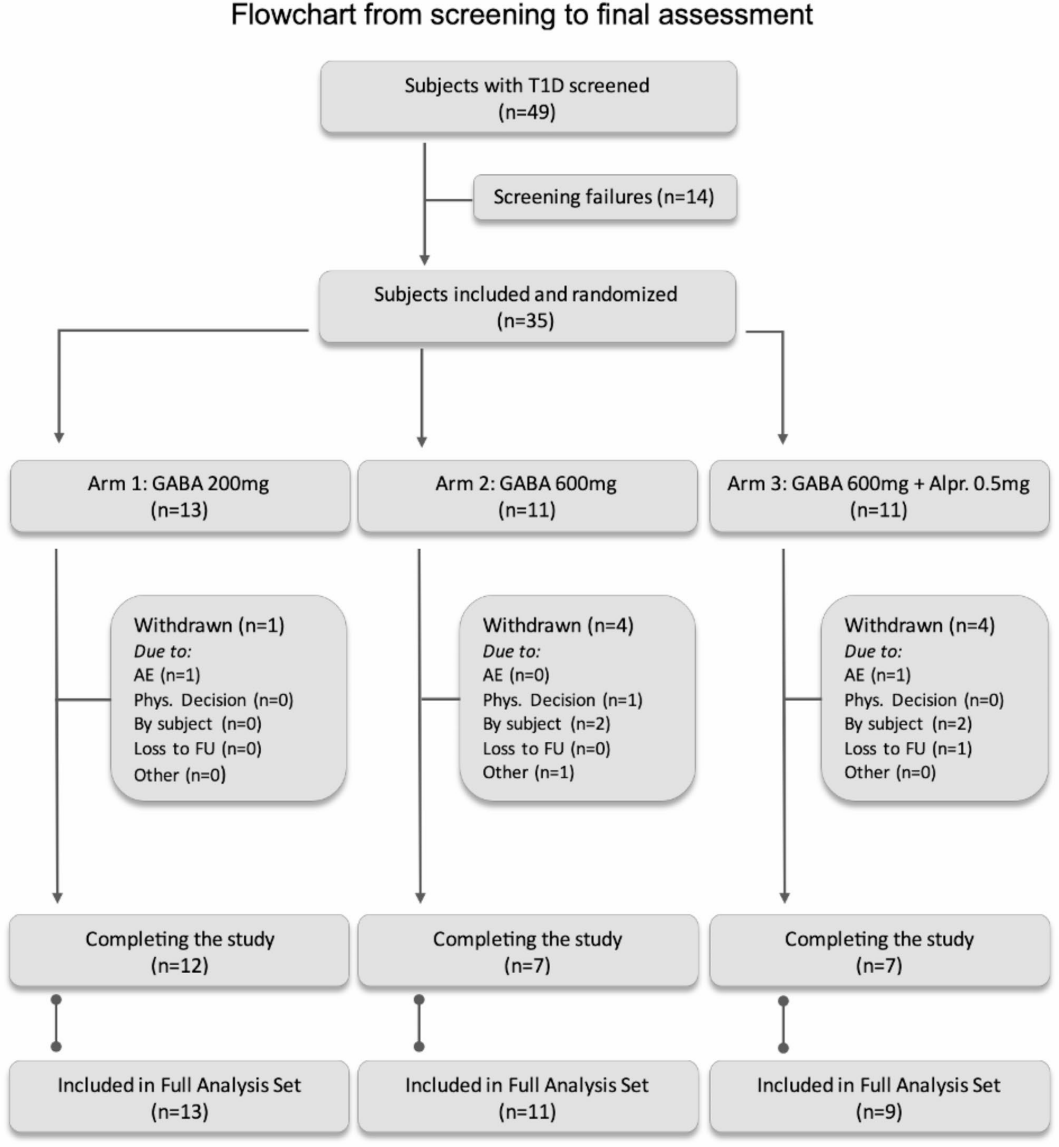
Flowchart of patient screening and inclusion. All subjects who were randomized and received at least one dose of GABA and had at least one post-baseline assessment along with a corresponding baseline measurement for any efficacy variable were included in the Full Analysis Set (*n* = 33).

Forty-nine male subjects with T1D were screened, of which 14 were screen failures. Thirty-five subjects were randomized (*Arm 1* *n* = 13; *Arm 2* *n* = 11; *Arm 3* *n* = 11), but two subjects in *Arm 3* never initiated the study drug due to withdrawal of informed consent by subject prior to the planned start (Fig. 2). The study arms (1–3) were comparable with regard to mean age, BMI and HbA1c (Table 1). In accordance with inclusion criteria, all subjects had a fasting C-peptide < 0.12 nmol/L. The C-peptide stratification between the study arms was based on detectable/undetectable C-peptide levels by clinical assay (cut off ≥0.05 nmol/L) and resulted in *n* = 2 (*Arm 1*), *n* = 1 (*Arm 2*) and *n* = 1 (*Arm 3*) C-peptide positive subjects. Based on the ultrasensitive C-peptide assay (cut-off ≥1.15 pmol/L) from the MMTT conducted at baseline there were *n* = 15 (*n* = 8 in *Arm1*, *n* = 3 in *Arm 2* and *n* = 4 in *Arm3*) subjects that had detectable C-peptide under fasting conditions and *n* = 27 subjects that had a detectable C-peptide level/levels at some time-point during the MMTT (*n* = 11 in *Arm 1*, *n* = 10 in *Arm 2* and *n* = 6 in *Arm3*).

**Table 1:** Participant characteristics at the screening visit.

Variable	GABA 200 mg (*N* = 13)	GABA 600 mg (*N* = 11)	GABA 600 mg + alprazolam 0.5 mg (*N* = 9)
Age (years)	33.5 ± 5.3	31.1 ± 7.6	27.4 ± 7.3
Sex (Male, n (%))	13 (100.0)	11 (100.0)	9 (100.0)
Height (cm)	182.4 ± 7.2	179.5 ± 6.6	182.2 ± 5.0
Weight (kg)	83.7 ± 13.3	80.3 ± 15.3	84.4 ± 14.8
Body mass index (kg/m^2^)	25.1 ± 3.4	24.9 ± 4.2	25.3 ± 4.0
Diastolic Blood Pressure (mmHg)	77.9 ± 7.0	76.6 ± 9.2	77.0 ± 6.1
Systolic Blood Pressure (mmHg)	125.3 ± 15.4	128.2 ± 15.9	131.4 ± 15.2
HbA1c (mmol/mol)	52.0 ± 8.9	54.0 ± 8.9	55.0 ± 11.1
Overall Vital Signs Evaluation. Abnormal, clinically significant, n (%)	0 (0)	1 (9.1)	0 (0)

Descriptive data of study subjects included in Full analysis set (*N* = 33), at screening visit. One subject in *Arm 2* was diagnosed with hypertension and started on antihypertensive treatment. Statistical comparisons were performed using either parametric methods (ANOVA) or nonparametric methods (Wilcoxon). Unless stated otherwise, data are presented as mean ± SEM, and p-values < 0.05 were considered statistically significant.

### Safety evaluation

Safety presentations are based on all randomized subjects who received at least one dose of GABA (*n* = 33).

#### Adverse events

Thirty out of the 33 subjects included in the safety population experienced AEs during the study. The majority of AEs were mild and only three patients had to discontinue or interrupt the study treatment because of an SAE/AE. The predominating symptoms classified as possibly or probably related to study treatment were from the gastrointestinal tract such as nausea and vomiting, general symptoms such as fatigue, symptoms from the nervous system such as dizziness, and an increase in aspartate aminotransferase (AST) *(Supplementary Table 1*).

Two serious adverse events (SAEs) were reported during the study, occurring in two different subjects in *Arm 1*. The first SAE was reported as a severe idiosyncratic liver reaction deemed probably related to the GABA treatment (AST and ALT values increased sevenfold but without giving rise to any symptoms). The subject was immediately discontinued from further treatment and the transaminase values normalized within a couple of days. The second SAE was a serious hypoglycemic event, according to the subject probably due to an overestimated bolus injection of rapid acting insulin and was hence reported as unlikely to be related to the study treatment. The subject fully regained from the hypoglycemic event without any sequelae.

In addition to the previously mentioned SAE, two AEs led to the discontinuation of study treatment. One subject in *Arm 2* received treatment with 600 mg GABA for 6 months at which time stomach pain evolved. The pain was deemed possibly related to study drug, and the subject was discontinued from treatment. The pain continued for 13 days after which it resolved. In *Arm 3*, one subject experienced two-to threefold elevated levels of AST compared to baseline for 16 days, which was likely related to 5 months of GABA treatment. This also resulted in the withdrawal of the study drug, whereafter normalization of AST levels followed.

A total of 9 subjects reported 17 events of elevated AST levels, deemed probably or possibly related to GABA treatment, where 2/33 (those mentioned above) were withdrawn from study treatment. For the other episodes, AST levels were only transiently increased and then normalized. There was no difference between the lower and higher concentrations of GABA, as three patients from each study group displayed increased AST levels at some point during the treatment.

Apart from instances of transaminase elevation, no other notable safety concerns were observed compared to baseline in terms of clinical chemistry, urine analysis, vital signs, physical examinations, neurological examinations or electrocardiography. A summary of AE/SAEs is presented in *Supplementary Table 1*.

#### Quality of life

A difference in quality of life, as measured by the RAND-36, was observed between *Arm 2* and *Arm 3* on day 180 and concerned the physical functioning category (i.e., whether health limits physical activities such as climbing stairs, walking or participating in sports) where subjects in *Arm 2* reported lower values than subjects in *Arm 3* (93.8 ± 6.4 vs. 99.3 ± 1.9 points, p *=* 0.0248). Additionally, lower self-reported emotional functioning (i.e., if emotional problems such as feeling depressed or anxious interfere with work or other activities) was observed in *Arm 1* compared to *Arm 3* on day 180 (69.4 ± 38.8 vs. 100 ± 0.0 points, p *=* 0.0351). However, these differences were not present at follow-up on day 210 (*Supplementary Table 2*).

### Efficacy evaluation

#### C-peptide

**Fig. 3:**
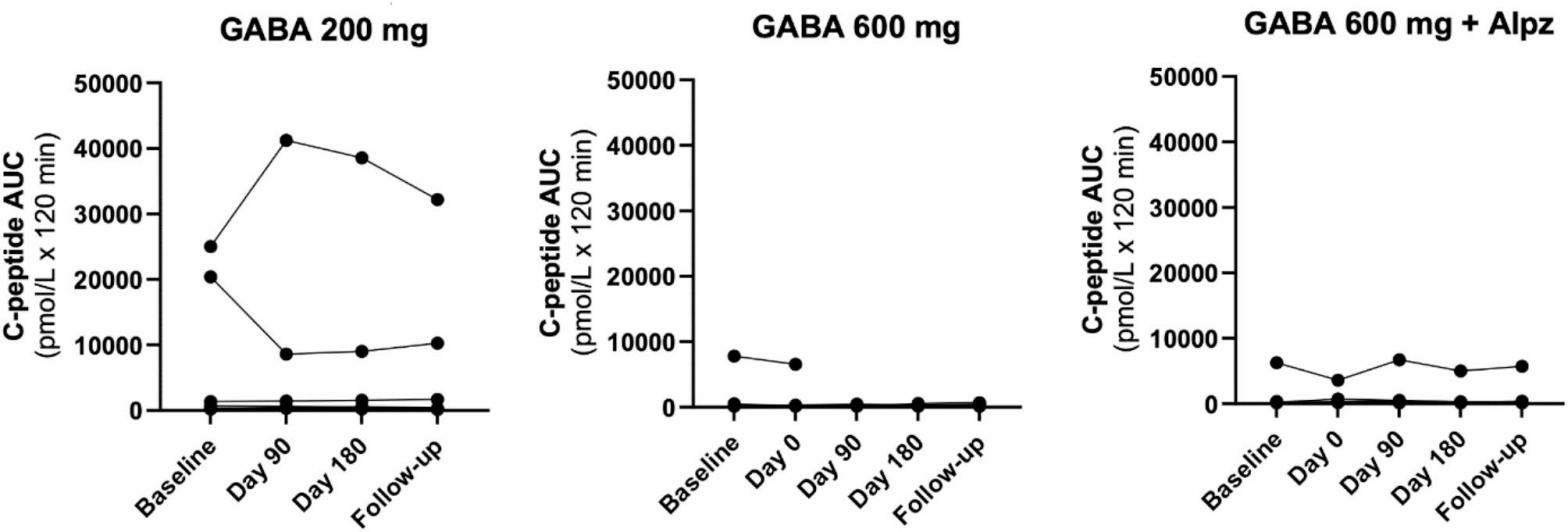
Differences in stimulated C-peptide in the treatment groups. Comparing differences in C-peptide (Area Under the Curve [AUC] mean 0–120 min) during a Mixed Meal Tolerance Test (MMTT) between baseline (Prior treatment) with 3 months of treatment, 6 months of treatment and the follow-up visit 1 month after termination of the study drug. In each graph single individual values connected by line are depicted. Mixed-effect analyses with Dunnett´s multiple comparison test was applied (p-values < 0.05 were to be considered statistically significant). No differences compared to baseline visit were observed in any of the treatment arms.

No effect on C-peptide levels were identified during the course of study when comparing fasting values, maximum values or AUC in response to MMTT, neither between groups nor within groups when comparing the different study visits to baseline (Fig. 3). This was true also when analyzing C-peptide with the ultrasensitive assay. Furthermore, no difference in C-peptide levels were observed in the individuals with detectable C-peptide at baseline. Similarly, there was no change in endogenous pro-insulin or insulin levels during the MMTT’s for any of the patients.

#### Continuous glucose monitoring and HbA1c

**Fig. 4:**
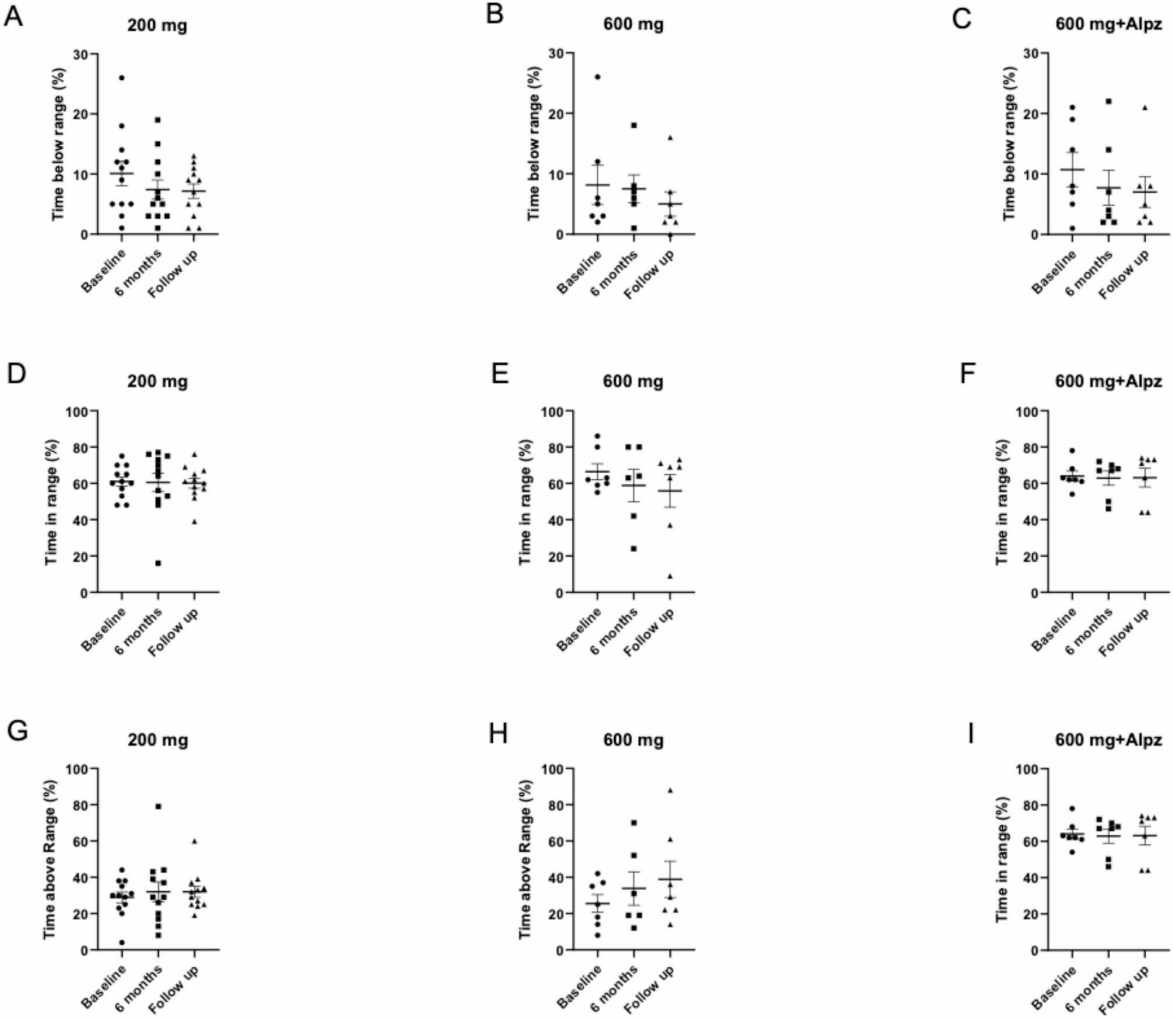
Comparison of continuous glucose monitoring (CGM)-data. Comparison of CGM metrics including % time below range (< 3.9 mmol/L) (**A**–**C**), % time in range 3.9-10 mmol/L) (**D**–**F**) and % time above range (> 10 mmol/L) (**G**–**I**) within each treatment group ranging from prior treatment (Baseline), 6 months of treatment to 1 month post treatment (Follow-up). A nonparametric one-way ANOVA with Dunn´s multiple comparisons test was applied. Results are presented as mean ± SEM, and p-values < 0.05 were considered statistically significant.

A total of 26 subjects completed the study and were included in the analysis of CGM data (*n* = 12 from *Arm 1*, and *n* = 7 from *Arms 2* and *3*, respectively). No differences in glucose level management concerning Time in range, Time below range or Time above range were identified during the Remygen^®^ treatment between groups or when compared to baseline values (Fig. 4).

**Fig. 5:**
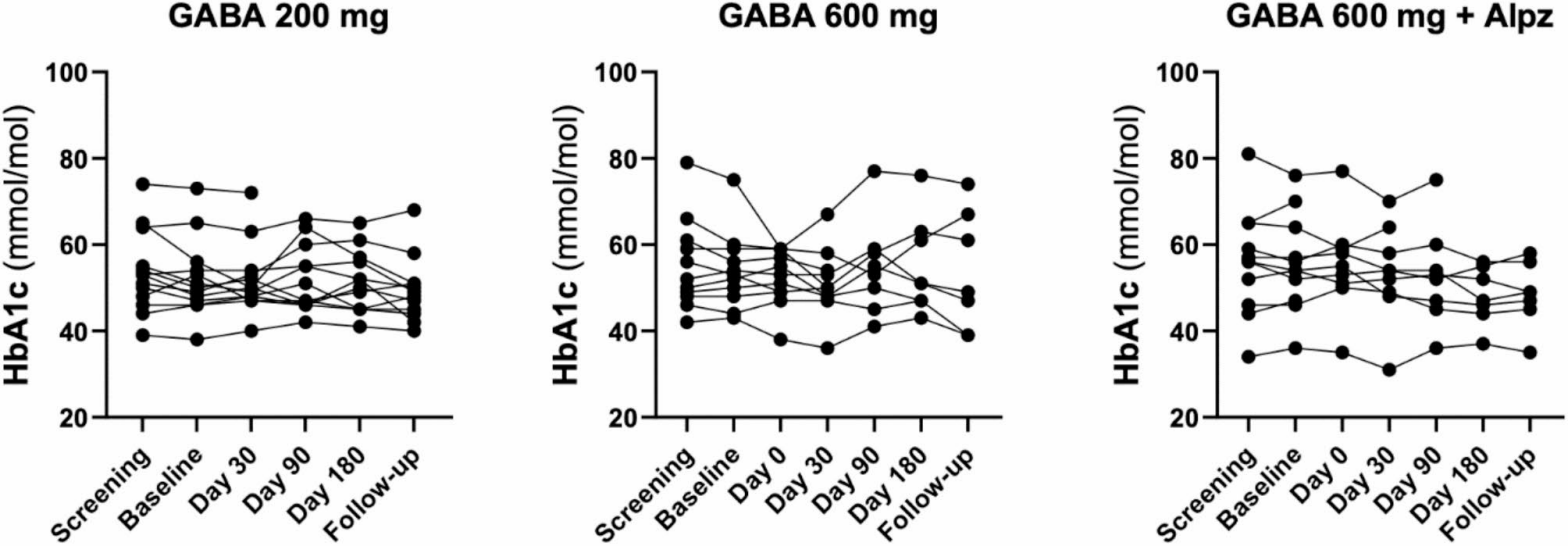
Comparison of HbA1c within the treatment groups. No differences were observed from the screening visit until follow-up, 1 month after study drug termination. Data are presented as individual graphs. All groups had a mean HbA1c below or close to the treatment target of 52 mmol/mol (7%) for subjects with T1D.

No difference in HbA1c was observed in any of the treatment arms from the baseline visit onwards. All groups had a mean HbA1c below or close to the treatment target of 52 mmol/mol (7%) for subjects with T1D (Fig. 5).

#### GABA-levels

**Fig. 6:**
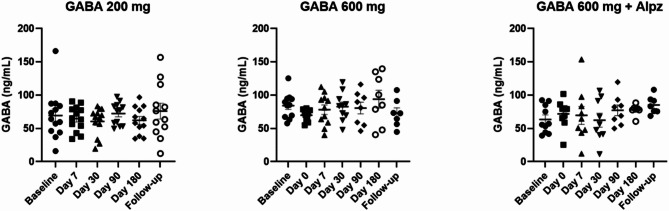
Plasma GABA levels within the treatment groups. Samples were collected as trough level, to measure the concentration of the drug immediately before the next dose. Mixed-effect analyses with Dunnett´s multiple comparison test was applied (p-values < 0.05 were to be considered statistically significant). No differences were observed from the Baseline visit onwards in any of the treatment arms, hence, no accumulation of the study-drug was observed over time.

Circulating levels of GABA were unaltered in the treatment arms from baseline visit to follow-up visit (Fig. 6).

#### Hypoglycemic clamp—counter-regulatory hormones

In contrast to our previous findings from the effect of short-term treatment with Remygen^®^^24^, we observed no differences in the delta counter regulatory hormonal response to induced hypoglycemia compared to baseline within each of the treatment arms.

## Discussion

This phase I/II, 3-arm, open-label, single-center randomized controlled trial was designed to investigate both the safety and the potential beta-cell regenerative effect of oral GABA therapy in subjects with long-standing T1D.

The main rationale was to test the possibility of GABA to regain beta-cell function in subjects with T1D in line with the previously reported experimental studies in animal models of T1D and on human islets^14,17,26,27^. In contrast to the previously published study by Martin et al. in which patients with new-onset T1D were treated with GABA^28^ our study targeted young adults with long-standing T1D (≥ five years). By this design, we tested the therapeutic effects of GABA as a potential beta-cell regenerative drug rather than its potential effects of limiting beta-cell death and decline of function. However, we observed no increase in either fasting- or stimulated C-peptide levels during the 6 months of GABA therapy. Nor did we observe any patients that converted from a C-peptide negative state, which also contradicts beta-cell neogenesis.

By sensitive C-peptide assays it has been shown that more than 70% of subjects with T1D for more than 5 years have remaining beta-cell function^5^. In the present study, we found that *n* = 15 subjects (43%) had measurable C-peptide under fasting conditions at baseline and *n* = 27 (77%) at least one of the time-points during the MMTT at baseline when C-peptide was analyzed with an ultrasensitive assay. Still, even in those with detectable C-peptide (i.e., some remaining beta-cells), there was no indication of a regain of beta-cell function during 6 months of GABA treatment which, although indirectly, contradicts any significant beta-cell regeneration in long-standing T1D.

No improvement in HbA1c levels was observed during the study treatment in any of the treatment arms. This aligns with the lack of effect on C-peptide concentrations and the unaltered time in range as measured by isCGM.

Although previous studies have found that the activation of GABA-A receptors by GABA or benzodiazepines can blunt the counter-regulatory hormonal response to hypoglycemia, our previous dose-escalation study demonstrated that Remygen^®^ acutely improved the counter-regulatory response^24^. In that study the number of patients was however limited and the treatment period was short (nine days) in contrast to the current study in which the GABA treatment was maintained for 6 months. In the current study, long-term treatment with GABA showed no effect on counter-regulatory hormones in response to hypoglycemia, but three out of four hypoglycemic clamps were performed 24 h after GABA intake, while in the former study the clamps were performed directly after GABA intake.

In this study, samples were collected before intake of Remygen^®^ as trough concentrations, while in the previous dose-escalation study GABA concentrations were measured as C-max and AUC, with increasing values as doses of GABA were elevated. Hence, the data of GABA concentrations in the present study demonstrate that there is no accumulation of the study-drug despite that it is a controlled-release formulation, which is advantageous from a safety perspective.

Two subjects in the study discontinued study treatment due to asymptomatically increased liver transaminase levels, and also some of the other individuals had transiently increased levels of AST and/or ALT. The exact cause(s) of the observed increases in AST and ALT levels is unknown. However, since the increases occurred in all treatment groups, a dose-dependent effect of the study drug is unlikely. Since this was a safety study, we discontinued study treatment in the two subjects with either steadily increasing transaminase levels or sustained increased transaminase levels for more than 14 days. It is therefore impossible to know if prolonged treatment would have caused symptomatic liver disease. In the rest of the cases, the transaminase increase was modest (up to twofold increased levels) and transient despite continuation of study drug intake. It is therefore possible that other causes than study drug intake caused these events, e.g. substantial physical exercise or alcohol intake, preferentially the former was also commonly reported by the study subjects. The observed GABA side effects on the liver are consistent with a previous study where subjects with cerebrovascular disorders were treated for 8 weeks with a daily dose of 3 g of GABA and transaminitis was described^29^ while the recent study by Martin et al. reported no such adverse events^28^. This discrepancy may be explained by age-dependent toxicity of GABA or simply that liver function and transaminases were not assessed in the latter study. Notably, despite the higher dose of GABA used in comparison to our trial no significant safety concerns were reported from this pediatric trial. GABA has previously been hypothesized to improve liver functions, as seen in a study on diabetic rats where GABA treatment resulted in decreased AST and ALT levels, an effect proposed to be the result of GABA minimizing oxidative stress in the liver^30^.

Other common AEs included gastrointestinal disorders and fatigue. However, regarding fatigue, no marked change was noted between study groups based on the RAND-36 questionnaire responses. A difference between the study groups was observed in physical functioning on day 180, between the GABA 600 mg group and the GABA 600 mg + 0.5 mg alprazolam group. Despite this difference, both groups reported very high levels of physical functioning, with scores of 93.8 and 99.3 points, respectively. Additionally on day 180, the GABA 200 mg group reported lower emotional functioning compared to the GABA 600 mg + 0.5 mg alprazolam. However, this difference was observed solely at this visit and was not a consistent difference between groups.

A limitation of the current trial was the lack of a placebo group, which makes it difficult to interpret especially the mild AEs reported. However, regarding the effects on C-peptide production each subject served as their own control, with results compared to baseline measurements. It cannot be excluded that a different pharmacokinetic profile or a higher dose of GABA might have led to a beta-cell regenerative effect. However, in the experimental studies in which beta-cell proliferation have been observed the doses used in most studies would convert to a human equivalent dose of 0.81–4.05 mg/kg^31,32^, i.e. lower than the doses used in our current study. Also, in experimental studies of transplanted human islets a beta-cell proliferative effect was observed already after 14-days of treatment (Tian et al. *Diabetes,* 2013). Furthermore, in the study of Martin et al. GABA was given twice daily, with a dose corresponding to up to 35 mg/kg, during a 12-month time period and no effect on C-peptide levels was observed. When considering the safety profile, a higher dose of Remygen^®^ would be challenging to use from a clinical perspective and based on the experimental data we find it unlikely that increasing the dose would be beneficial based on the doses used in experimental studies. However, it cannot be ruled out that GABA in combination with other drugs, such as DPP-IV inhibitors, could have a beta-cell proliferative effect which have been found in more recent experimental studies^33,34^. Also, in a retrospective clinical observational study it has been found that a combination treatment of GABA, a DPP-IV inhibitor and a proton pump inhibitor improved glycemic control and increased insulin secretion in adult patients with new onset T1D^35^. However, it should be noted that this effect could also be unrelated to GABA and merely reflect the stimulating effect of DPP-IV inhibitors since these agents are known to have beneficial effects in new-onset T1D in adults with the capacity to reduce or even temporarily deplete the need for exogenous insulin therapy^36^.

## Conclusion

Although experimental studies have shown promising results, our 6-month treatment clinical trial indicates that GABA lacks the effect to regain beta-cell function in adult subjects with long-standing T1D. In addition, side effects such as increased liver transaminase levels were commonly observed.

## Electronic supplementary material

Below is the link to the electronic supplementary material.

Supplementary Material 1

Supplementary Material 2

## Data Availability

Data will be made available upon reasonable request. Requests should be directed to the corresponding author. The study protocol is available online (https://clinicaltrials.gov/study/NCT03635437).
