# Supplementary material for: Long-term gamma-aminobutyric acid (GABA) treatment fails to regain beta-cell function in longstanding type 1 diabetes in a randomized trial
Source: Sci Rep. 2025 Apr 4;15:11530. doi: 10.1038/s41598-025-95751-y (PMC11971400; doi:10.1038/s41598-025-95751-y)
Supplement: Supplementary file 1 — Supplementary Material 1 [file 41598_2025_95751_MOESM1_ESM.docx]

**Supplementary table 1.** Overall summary of adverse events.

Thirty out of the thirty-three patients included in the safety population experienced AEs during the study. The majority of AEs were mild and only three patients had to discontinue or interrupt the study treatment because of an AE.

|  | **GABA 200mg/day, n** | **GABA 600mg/day, n** | **GABA 600mg/day**  **+**  **Alprazolam 0.5mg, n** | **Total, n** |
| --- | --- | --- | --- | --- |
| Number of patients in Safety Set | 13 | 11 | 9 | 33 |
| Number of patients reporting any AE (number of AEs) | 11 (32) | 10 (33) | 9 (41) | 30 (106) |
| Number of patients reporting any SAE (number of SAEs) | 2 (2) | 0 | 0 | 2 (2) |
| Number of patients reporting an AE leading to drug discontinuation (number of AEs) | 1 (2) | 0 | 1 (1) | 2 (3) |
| Number of patients reporting an AE leading to drug interruption (number of AEs) | 0 | 1 (1) | 0 | 1 (1) |
| Number of patients reporting any AE possibly related to study treatment (number of AEs) | 6 (12) | 4 (13) | 8 (22) | 18 (47) |
| Number of patients reporting any AE probably related to study treatment (number of AEs) | 1 (2) | 4 (7) | 2 (3) | 7 (12) |
| Number of patients reporting any AE possibly or probably related to study treatment (number of AEs) | 7 (14) | 7 (20) | 9 (25) | 23 (59) |
| Number of unlikely related AEs | 3 | 6 | 5 | 14 |
| Number of not related AEs | 15 | 7 | 5 | 27 |
| Number of patients reporting any AE with intensity = Severe (number of AEs) | 1 (1) | 0 | 0 | 1 (1) |
| Number of patients reporting any AE with intensity = Moderate (number of AEs) | 2 (2) | 1 (1) | 1 (1) | 4 (4) |
| Number of patients reporting any AE with intensity = Mild (number of AEs) | 11 (29) | 10 (32) | 9 (40) | 30 (101) |
| Number of patients reporting any AE with maximum intensity = Moderate | 2 | 1 | 1 | 4 |
| Number of patients reporting only Mild AEs | 8 | 9 | 8 | 25 |
