# Supplementary material for: Long-term gamma-aminobutyric acid (GABA) treatment fails to regain beta-cell function in longstanding type 1 diabetes in a randomized trial
Source: Sci Rep. 2025 Apr 4;15:11530. doi: 10.1038/s41598-025-95751-y (PMC11971400; doi:10.1038/s41598-025-95751-y)
Supplement: Supplementary file 2 — Supplementary Material 2 [file 41598_2025_95751_MOESM2_ESM.docx]

**Supplementary table 2.** RAND-36 questionnaire comparing 6 months of treatment and Follow-up visit, 1 month after treatment termination.

Quality of Life (QoL) was assessed with the Diabetes treatment satisfaction questionnaire (DTSQ) (*see supplementary table 3*) and the RAND-36 questionnaire at Baseline visit, at the last day of study drug (6 months), at the Follow-up visit and 1 month post treatment (for arms 2 and 3 an additional test was performed at treatment start). The only difference in quality of life, as measured by the RAND-36, was observed between *Arm 2* (GABA 600mg) and *Arm 3* (GABA 600mg + Alprazolam 0.5mg) on day 180 concerning physical functioning, where subjects in *Arm 2* reported lower values than subjects in *Arm 3*. Additionally, lower self-reported emotional functioning was observed in *Arm 1* compared to *Arm 3* on day 180. However, these differences were not present at follow-up on day 210. P-values <0.05 were considered statistically significant.

*Based on the non-parametric Mann-Whitney test.

**Hodges-Lehmann is the estimator of the location shift or the estimator of the median of differences. The second group was used as reference.

| **RAND-36 domain** | Visit |  | n_1_ | n_2_ | Hodges-Lehmann location shift estimate (95% CI)** | P-value* |
| --- | --- | --- | --- | --- | --- | --- |
| Physical functioning | Day 180 | GABA 200 mg vs. GABA 600 mg | 12 | 8 | 5.0 (0.0 ; 5.0) | 0.0664 |
|  |  | GABA 200 mg vs. GABA 600 mg + Alprazolam 0.5 mg | 12 | 7 | 0.0 (0.0 ; 0.0) | 0.5529 |
|  |  | GABA 600 mg vs. GABA 600 mg + Alprazolam 0.5 mg | 8 | 7 | -5.0 (-10.0 ; 0.0) | **0.0248*** |
|  | Day 210 | GABA 200 mg vs. GABA 600 mg | 12 | 7 | 2.5 (0.0 ; 10.0) | 0.1704 |
|  |  | GABA 200 mg vs. GABA 600 mg + Alprazolam 0.5 mg | 12 | 7 | 0.0 (0.0 ; 0.0) | 0.6773 |
|  |  | GABA 600 mg vs. GABA 600 mg + Alprazolam 0.5 mg | 7 | 7 | -5.0 (-15.0 ; 0.0) | 0.1359 |
| Role functioning/physical | Day 180 | GABA 200 mg vs. GABA 600 mg | 12 | 8 | 0.0 (0.0 ; 0.0) | 0.2667 |
|  |  | GABA 200 mg vs. GABA 600 mg + Alprazolam 0.5 mg | 12 | 7 | 0.0 (0.0 ; 0.0) | 0.8418 |
|  |  | GABA 600 mg vs. GABA 600 mg + Alprazolam 0.5 mg | 8 | 7 | 0.0 (0.0 ; 0.0) | 0.3496 |
|  | Day 210 | GABA 200 mg vs. GABA 600 mg | 12 | 7 | 0.0 (0.0 ; 0.0) | 0.6773 |
|  |  | GABA 200 mg vs. GABA 600 mg + Alprazolam 0.5 mg | 12 | 7 | 0.0 (0.0 ; 25.0) | 0.4110 |
|  |  | GABA 600 mg vs. GABA 600 mg + Alprazolam 0.5 mg | 7 | 7 | 0.0 (0.0 ; 50.0) | 0.2974 |
| Role functioning/emotional | Day 180 | GABA 200 mg vs. GABA 600 mg | 12 | 8 | 0.0 (-33.3 ; 0.0) | 0.3349 |
|  |  | GABA 200 mg vs. GABA 600 mg + Alprazolam 0.5 mg | 12 | 7 | -16.7 (-33.3 ; 0.0) | **0.0351*** |
|  |  | GABA 600 mg vs. GABA 600 mg + Alprazolam 0.5 mg | 8 | 7 | 0.0 (0.0 ; 0.0) | 0.2036 |
|  | Day 210 | GABA 200 mg vs. GABA 600 mg | 12 | 7 | 0.0 (-33.3 ; 0.0) | 0.3808 |
|  |  | GABA 200 mg vs. GABA 600 mg + Alprazolam 0.5 mg | 12 | 7 | 0.0 (-33.3 ; 33.3) | 0.8856 |
|  |  | GABA 600 mg vs. GABA 600 mg + Alprazolam 0.5 mg | 7 | 7 | 0.0 (0.0 ; 33.3) | 0.3771 |
| Energy/fatigue | Day 180 | GABA 200 mg vs. GABA 600 mg | 12 | 8 | -5.0 (-35.0 ; 10.0) | 0.3936 |
|  |  | GABA 200 mg vs. GABA 600 mg + Alprazolam 0.5 mg | 12 | 7 | 5.0 (-20.0 ; 20.0) | 0.7663 |
|  |  | GABA 600 mg vs. GABA 600 mg + Alprazolam 0.5 mg | 8 | 7 | 10.0 (-10.0 ; 30.0) | 0.2218 |
|  | Day 210 | GABA 200 mg vs. GABA 600 mg | 12 | 7 | 0.0 (-30.0 ; 25.0) | 0.9661 |
|  |  | GABA 200 mg vs. GABA 600 mg + Alprazolam 0.5 mg | 12 | 7 | -2.5 (-25.0 ; 20.0) | 0.8319 |
|  |  | GABA 600 mg vs. GABA 600 mg + Alprazolam 0.5 mg | 7 | 7 | 0.0 (-25.0 ; 30.0) | 0.8969 |
| Emotional well-being | Day 180 | GABA 200 mg vs. GABA 600 mg | 12 | 8 | -4.0 (-20.0 ; 16.0) | 0.7856 |
|  |  | GABA 200 mg vs. GABA 600 mg + Alprazolam 0.5 mg | 12 | 7 | 0.0 (-12.0 ; 8.0) | 0.9315 |
|  |  | GABA 600 mg vs. GABA 600 mg + Alprazolam 0.5 mg | 8 | 7 | 0.0 (-16.0 ; 16.0) | 1.0000 |
|  | Day 210 | GABA 200 mg vs. GABA 600 mg | 12 | 7 | -4.0 (-20.0 ; 16.0) | 0.6710 |
|  |  | GABA 200 mg vs. GABA 600 mg + Alprazolam 0.5 mg | 12 | 7 | 0.0 (-12.0 ; 12.0) | 0.9661 |
|  |  | GABA 600 mg vs. GABA 600 mg + Alprazolam 0.5 mg | 7 | 7 | 4.0 (-16.0 ; 16.0) | 0.5201 |
| Social functioning | Day 180 | GABA 200 mg vs. GABA 600 mg | 12 | 8 | 0.0 (-12.5 ; 0.0) | 0.6936 |
|  |  | GABA 200 mg vs. GABA 600 mg + Alprazolam 0.5 mg | 12 | 7 | 0.0 (-12.5 ; 0.0) | 0.1992 |
|  |  | GABA 600 mg vs. GABA 600 mg + Alprazolam 0.5 mg | 8 | 7 | 0.0 (-12.5 ; 0.0) | 0.3315 |
|  | Day 210 | GABA 200 mg vs. GABA 600 mg | 12 | 7 | 0.0 (0.0 ; 12.5) | 0.2209 |
|  |  | GABA 200 mg vs. GABA 600 mg + Alprazolam 0.5 mg | 12 | 7 | 0.0 (0.0 ; 12.5) | 0.4417 |
|  |  | GABA 600 mg vs. GABA 600 mg + Alprazolam 0.5 mg | 7 | 7 | 0.0 (-25.0 ; 12.5) | 0.8368 |
| Pain | Day 180 | GABA 200 mg vs. GABA 600 mg | 12 | 8 | 10.0 (0.0 ; 32.5) | 0.1092 |
|  |  | GABA 200 mg vs. GABA 600 mg + Alprazolam 0.5 mg | 12 | 7 | 0.0 (-12.5 ; 10.0) | 0.6467 |
|  |  | GABA 600 mg vs. GABA 600 mg + Alprazolam 0.5 mg | 8 | 7 | -10.0 (-32.5 ; 10.0) | 0.2324 |
|  | Day 210 | GABA 200 mg vs. GABA 600 mg | 12 | 7 | 0.0 (-10.0 ; 32.5) | 0.5349 |
|  |  | GABA 200 mg vs. GABA 600 mg + Alprazolam 0.5 mg | 12 | 7 | 1.3 (-10.0 ; 22.5) | 0.4276 |
|  |  | GABA 600 mg vs. GABA 600 mg + Alprazolam 0.5 mg | 7 | 7 | 0.0 (-32.5 ; 22.5) | 1.0000 |
| General health | Day 180 | GABA 200 mg vs. GABA 600 mg | 12 | 8 | 10.0 (-10.0 ; 30.0) | 0.3321 |
|  |  | GABA 200 mg vs. GABA 600 mg + Alprazolam 0.5 mg | 12 | 7 | 0.0 (-20.0 ; 10.0) | 0.7308 |
|  |  | GABA 600 mg vs. GABA 600 mg + Alprazolam 0.5 mg | 8 | 7 | -15.0 (-35.0 ; 5.0) | 0.2214 |
|  | Day 210 | GABA 200 mg vs. GABA 600 mg | 12 | 7 | 20.0 (-5.0 ; 40.0) | 0.1157 |
|  |  | GABA 200 mg vs. GABA 600 mg + Alprazolam 0.5 mg | 12 | 7 | 0.0 (-20.0 ; 15.0) | 0.9319 |
|  |  | GABA 600 mg vs. GABA 600 mg + Alprazolam 0.5 mg | 7 | 7 | -25.0 (-45.0 ; 5.0) | 0.0952 |
